# Insights into the stereospecificity of the d-specific dehalogenase from *Rhizobium* sp. RC1 toward d- and l-2-chloropropionate

**DOI:** 10.1080/13102818.2014.937907

**Published:** 2014-10-23

**Authors:** Ismaila Yada Sudi, Azzmer Azzar Abdul Hamid, Mohd Shahir Shamsir, Haryati Jamaluddin, Roswanira Abdul Wahab, Fahrul Huyop

**Affiliations:** ^a^ Faculty of Biosciences and Medical Engineering (FBME), Universiti Teknologi Malaysia, Johor Bahru, Johor, Malaysia; ^b^ Faculty of Science, International Islamic University Malaysia, Kuantan, Pahang, Malaysia; ^c^ Faculty of Science, Universiti Teknologi Malaysia, Johor Bahru, Johor, Malaysia

**Keywords:** binding energy, d-specific dehalogenase, d-2-chloropropionate, dehalogenase, interacting residue, hydrogen-bond length, stereospecificity

## Abstract

Halogenated compounds are recalcitrant environmental pollutants prevalent in agricultural fields, waste waters and industrial by-products, but they can be degraded by dehalogenase-containing microbes. Notably, 2-haloalkanoic acid dehalogenases are employed to resolve optically active chloropropionates, as exemplified by the d-specific dehalogenase from *Rhizobium* sp. RCI (DehD), which acts on d-2-chloropropionate but not on its l-enantiomer. The catalytic residues of this dehalogenase responsible for its affinity toward d-2-chloropropionate have not been experimentally determined, although its three-dimensional crystal structure has been solved. For this study, we performed *in silico* docking and molecular dynamic simulations of complexes formed by this dehalogenase and d- or l-2-chloropropionate. Arg134 of the enzyme plays the key role in the stereospecific binding and Arg16 is in a position that would allow it to activate a water molecule for hydrolytic attack on the d-2-chloropropionate chiral carbon for release of the halide ion to yield l-2-hydroxypropionate. We propose that within the DehD active site, the NH group of Arg134 can form a hydrogen bond with the carboxylate of d-2-chloropropionate with a strength of ∼4 kcal/mol that may act as an acid–base catalyst, whereas, when l-2-chloropropionate is present, this bond cannot be formed. The significance of the present work is vital for rational design of this dehalogenase in order to confirm the involvement of Arg16 and Arg134 residues implicated in hydrolysis and binding of d-2-chloropropionate in the active site of d-specific dehalogenase from *Rhizobium* sp. RC1.

## Abbreviations

2CP: 2-chloropropionate
d-2CP: d-2-chhloropropionate
l-2CP: l-2-chloropropionate
DehD: d-specific dehalogenase from *Rhizobium* sp.RC1
HadD: d-specific dehalogenase from *Pseudomonas putida* AJ1
MD: molecular dynamics
d-2CP–DehD: a complex of d-2CP with DehD protein
l-2CP–DehD: a complex of l-2CP with DehD protein

## Introduction

Many naturally occurring organohalogens exist, including 2CP,[1] which is stable and toxic, and has been found in microbes, insects, marine plants and animals, including humans.[2] Microbial dehalogenases can convert xenobiotic compounds into their enantiomeric products.[3,4] The subject of the study reported herein, the DehD, along with other phylogenetically related but non-stereoselective dehalogenases, has been categorized as group I dehalogenase (DehI).[5] Among these enzymes, the best characterized is the non-stereoselective haloacid dehalogenase from *Pseudomonas putida* strain 113, which has a single active site and converts both d- and l-2-haloalkanoic acids into their enantiomeric configurations.[6,7] The sequence of the non-stereoselective haloacid dehalogenase from *Pseudomas putida* strain 113 is similar to that of the HadD,[8] which specifically inverts the configuration of the C2 atom of d-2-haloalkanoic acids to yield l-2**-**hydroxyalkanoic acids.[4] Conversely, the sequence of the HadD shares only 23% similarity with the DehD and 15% similarity with the non-stereospecific dehalogenase from *Pseudomonas putida* PP3 for which a three-dimensional structure has been elucidated.[9]

Dehalogenases that act on d-2CP have the potential to be used for the detoxification of the environmental pollutant, d-2CP,[4,10] to produce l-lactics (polylactides) for medicinal purposes,[11–13] and to produce optically pure l-2CP from a racemic mixture for the production of the phenoxy herbicide Fusilade,[14] which is commercially manufactured by Zeneca (Imperical Chemical Industries, Plc.).[4] In addition, the utility of the l-2-haloacid dehalogenase (DehCI) from *Pseudomonas* CBS32 to produce optically pure d-chloropropionic acid has been demonstrated.[15]

The relationship between the physicochemical properties of an enzyme active site and its substrate(s)/product(s) underlies enzymatic specificity.[16] For example, an enzyme with a hydrogen-bond-donating group requires a substrate with a hydrogen-bond-accepting group, or a charged catalytic residue requires an oppositely charged substrate moiety. In general, an enzyme and its substrate initially form a non-covalent Michaelis–Menten-type enzyme–substrate complex before catalysis occurs.[17] The binding energies of the haloalkane dehalogenase (DhIA) from *Xanthobacter autotrophicus* GJ10 and various substrates, which might serve as mimics of Michaelis–Menten-type complexes, have been calculated after performing *in situ* docking experiments.[18] However, to the best of our knowledge, such calculations have not been made for DehD. Therefore, for the work reported herein, we calculated the binding energies of d-2CP and l-2CP individually complexed with DehD and examined the orientations of d-2CP and l-2CP in the DehD active site after the docking and MD studies to gain insight into the substrate selectivity of the enzyme.

## Materials and methods

**Figure 1.**
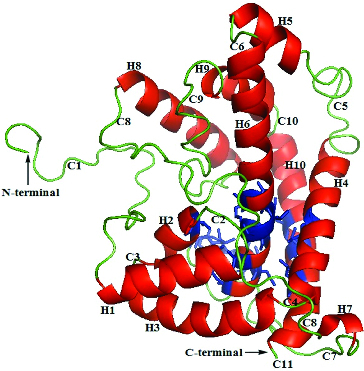
Three-dimensional structure of DehD [19]. The binding-site residues (Val45, Met79, Ala130, Thr131, Val132, Ser133, Arg134, Tyr135, Leu136, Gln138, Asp139, Ala145, Ile147, Ile148, His149, Leu150, Leu151, Ala250, Cys253 and Leu257), 14 residues (Val45, Met79, Ala130, Thr131, Val132, Ser133, Arg134, Tyr135, Leu136, Gln138, Asp139, Ala250,Cys253 and Leu257) are shown in blue. The helices are labelled H1–H10, and the loops are labelled C1–C11.Note: Figure 1 is a reprint of a figure previously published by MDPI-Sudi et al., (2012) Structure prediction, molecular dynamics simulation and docking studies of D-specific dehalogenase from Rhizobium sp. RCI. Int J Mol Sci. 13:15724–15754. published by MDPI.

**Figure 2.**
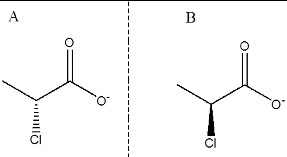
Substrate structures. (**A**) d-2CP ((2R)-2-chloropropionate) and (**B**) l-2CP ((2S)-2-chloropropionate).

The three-dimensional model of DehD from *Rhizobium* sp. RCI [19] (Figure 1) was used, and d- and l-2CP from the PubChem structures (http://pubchem.ncbi.nlm.nih.gov) for the docking exercises (Figure 2). AutoDock 4.2 [20] was used to separately dock DehD with d- or l-2-CP. Polar hydrogen atoms and Kollman and Gasteiger charges [6] were added using AutoDockTools [21] to DehD and each substrate. To accommodate the active-site residues, the grid spacing was adjusted from 0.375 Å (default) to 1 Å spacing, the grid box dimensions were also adjusted to 22 × 24 × 38 points and the *x*, *y*, *z* dimensions to 60.711, 69.395, 64.414 points, using Autogrid.[20] The grid space contained 22,425 points after uploading the affinity maps so that they could be visualized on the graphical user interface. Lamarckian genetic algorithm parameters [20] were employed for 100 substrate-docking runs, each with a population size of 150 individuals, a maximum number of 2.5 × 10^6^ energy evaluations, 27,000 maximum generations, an elitism value of 1, a mutation rate of 0.02, a crossover rate of 0.80, and a variance of Cauchy distribution for gene mutation of 1.00; the number of generations for picking the worst individual was 10.00.[21] The docking results were analysed after setting the clustering tolerance to 0.5, 1.0 and 2.0. The best conformation with a root-mean-square tolerance of 1.0 was selected for the d- and l-2CP-containing complexes. The docked outputs were saved in protein data bank (PDB); partial charge and atom type format (PDBQT) coordinate structure files for further analysis and conversion into PDB files for MD simulation studies.

## Molecular dynamics simulations of the DehD–substrate complexes

All MD simulations were performed by GROMACS 4.6.3 [22] run on a Ubuntu 1.6 GHz computer with a quad core processor. The protein–ligand complex PDB files obtained from docking runs for the DehD were simulated by MD simulations for affinity studies. The protein**–**ligand complexes in PDBQT file formats were converted into PDB files, which were then separated into protein and ligand coordinate files. The topology of DehD was determined using the GROMOS96 53a6 force field values [23] and those for d- and l-2CP were prepared using PRODRG,[24] which were subsequently included as part of the complex topologies. The simulation box was cubic with its edges ∼1.0 nm from the periphery of the complexed molecules. The entire box was solvated with 17,838 space water molecules. The system energy was minimized for molecular stabilization and remove bad van der Waals contacts at a temperature of 300 K, pressure of 798 mmHg and density of 1000 kg m^−3^; maintaining the periodic boundaries at 10 ns (nanoseconds). During the equilibration process, the substrates were restrained within the DehD active site. The energy-minimized DehD complexed with substrate structures were merged and the equilibrium protein-non-protein effects were obtained using the Particle Mesh Ewald method and the appropriate Coulomb potential.[25,26] The positional restraints complexes were released after 5 × 10^6^ equilibration steps at 300 K for every 0.5 ps (picoseconds). Then the MD simulations were run for 10 ns. The MD simulations were used to determine the Cα root-mean-square-deviation (RMSD) values, the root-mean-square-fluctuation (RMSF) values of the α-carbon atoms, the system energies and the radii of gyration and to identify any possible interactions between active-site residues and the substrates. Plots were graphed using the GRraphing Advanced Computational Exploration of data (Xmgr), a two-dimensional plotting tool (http://plasma-gate.weizmann.ac.il/Grace/).

## Results and discussion

### DehD binding affinity toward d- and l-2CP

**Figure 3.**
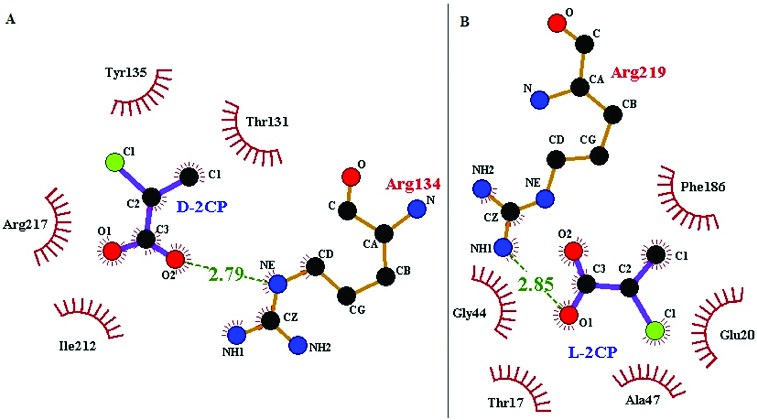
Two-dimensional diagrams of the DehD residues that interact with d-2CP (**A**) and l-2CP (**B**). Colour coding: nitrogen, blue; oxygen, red; carbon, black; and chlorine, green. d-2CP and l-2CP bonds are shown in purple; non-substrate bonds are shown in orange; hydrogen bonds are shown in olive green and their lengths are provided; DehD residues that form hydrophobic interactions between DehD [34] and d-2CP and l-2CP are shown as red spikes, and their atoms involved are shown as balls.

**Figure 4.**
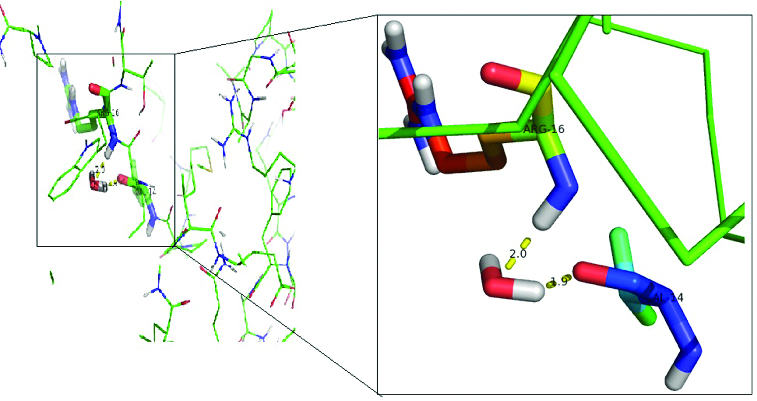
Interaction of the activated water molecule with DehD active-site residues. The insert shows an enlarged image of the Val14 and Arg16 interactions with a water molecule.

Examination of the docked d-2CP–DehD complex (Figure 3) revealed that the NE of Arg134 and a carboxyl oxygen of d-2CP are within hydrogen-bonding distance from each other (the length and angle between NE of Arg134 and the carboxyl oxygen of d-2CP are 2.79 Å and 110º, respectively). The side chains of four residues, Ile212, Arg217, Tyr135 and Thr131, were observed to make hydrophobic interactions with d-2CP which is hydrogen bonded to Arg134. Similarly, Ile212 and Arg217 were very close residues to the carboxyl group of d-2CP. In addition, water molecule showed an apparent interaction with DehD via Val14 and Arg16 (Figure 4). The interaction of a water molecule with valine and arginine in DehD may be an activation of the amino-acid residues for hydrolytic catalysis. For the l-2CP–DehD complex, a hydrogen bond is possible, involving the NH1 of Arg219 and carboxyl oxygen of l-2CP with a length of 2.85 Å and an angle of ∼171º. In addition, the side chains of five DehD residues, Gly44, Thr17, Ala47, Glu20 and Phe186, are in close contact with l-2CP. Of all these five residues in hydrophobic interaction with l-2CP, valine and arginine were not involved and hence the significance of their interaction with water molecule in DehD could not be of relevance to l-2CP and DehD hydrolytic interaction. The proposed hydrogen-bond interactions between the arginines of DehD and the carboxyl oxygens of d-2CP and l-2CP are considered to be of moderate strength,[27] with lengths [28,29] and angles [30] suitable for effective protein–substrate hydrogen bonding. To strengthen the proposal that a hydrogen bond is formed between the NE hydrogen of Arg134 or Arg219 and the carboxyl oxygen of d- or l-2CP, respectively, we performed MD simulations of the docked structures. The calculated docking binding energies for the d-2CP–DehD and l-2CP–DehD complexes are 4.11 and 4.18 kcal/mol, respectively (Table 1). After MD energy minimization, the total energy values of the systems are –1.51 × 10^5^ and –1.52 × 10^5^ kcal/mol, respectively, for the d- and l-2CP complexes. In the absence of experimental values for the binding energies involving any DehI or mutant of a DehI with a substrate, we compared our calculated binding energies with the experimentally derived free energies of binding for 4-chlorobenzoyl-coenzyme A dehalogenase mutants complexed with 4-chlorobenzoic acid, which are ∼1.3–5.1 kcal/mol.[31] The calculated binding energies of 4.11 and 4.18 kcal/mol for the d-2CP–DehD protein and l-2CP–DehD protein complexes, respectively, are within the aforementioned range of energies. This binding energy decreased after energy minimization by 0.63% to give a stable protein conformation, which suggests that DehD protein preferentially binds to d-2CP than to l-2CP.

**Table 1.** Binding energies of DehD complexed with d- and l-2-chloropropionates.

Energy parameter (kcal/mol)	d-2CP–DehD complex	l-2CP–DehD complex
Binding energy	4.11	4.18
Total energy after MD simulations	−1.51 × 10^5^	−1.52 × 10^5^
Intermolecular energy	−3.65	−3.57
van der Waals interactions	−1.87	−1.91
Electrostatic interactions	−1.78	−1.66
Total energy	0.12	0.17

Note: The values are from the docking results.

**Figure 5.**
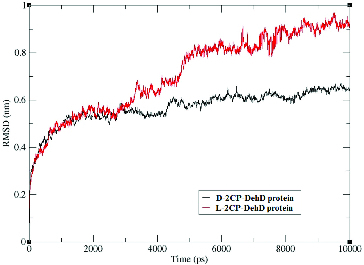
Root-mean-square deviations of the DehD [34] Cα atoms vs. time for the d-2CP–DehD and l-2CP–DehD complexes. d-2CP–DehD (black); l-2CP-DehD (red).

Time-dependent Cα RMSD values were obtained during the 10,000-ps MD simulations (Figure 5). Plots of the standard deviations of the Cα RMSDs of the complexes are shown in Figure 4 and suggest that the complexes are stable after MD simulation. The average Cα RMSD for DehD complexed with d-2CP is 0.6 nm ± 0.1 nm, whereas that for DehD complexes with l-2CP is 0.7 nm ± 0.2 nm. Both complexes were considered to be stable by 9500 ps, even though the average Cα RMSD value for the l-2CP complexed with DehD is greater than that for the d-2CP complexed with DehD. When the simulation of the d-2CP complex simulation was considered alongside the simulation of l-2CP–DehD protein complex structures, the former had a more stable structure than the later structure. Because the average Cα RMSD for DehD complexed with d-2CP is smaller than that for the l-2CP–DehD protein complex, we consider the former complex to be more stable than the latter complex, which may be reflected in part in the binding energy.

**Figure 6.**
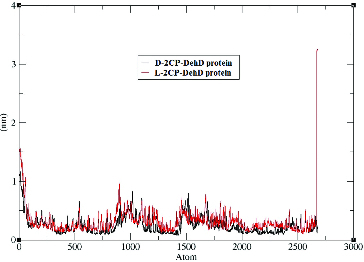
Root-mean-square fluctuations of the DehD Cα atoms vs. the number of atoms for the d-2CP–DehD and l-2CP–DehD complexes. d-2CP–DehD (black); l-2CP–DehD (red).

The Cα RMSF values derived from the MD simulations for the d–2CP–DehD protein and l-2CP–DehD protein complexes were calculated to assess backbone flexibility (Figure 6). For the d-2CP–DehD protein complex, the RMSF is 0.25 nm ± 0.16 nm, and for the l-2CP–DehD protein complex, it is 0.31 nm ± 0.23 nm. This suggests that there is no significant difference between the two complexes even though the atoms of the carboxy terminal residue Pro265 of both complexes fluctuated to a greater extent, ∼3.25 nm. The calculated RMSF value for the d-2CP complex is 48.39% smaller than that for the l-2CP–DehD complex, suggesting that the d-2CP–DehD complex is more stable.

**Figure 7.**
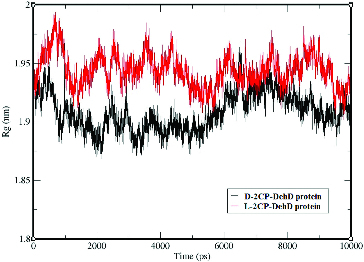
The radii of gyration vs. time for the d-2CP–DehD and l-2CP–DehD complexes. d-2CP–DehD (black); l-2CP–DehD (red).

The average radius of gyration for the d-2CP–DehD protein complex is 1.91 nm ± 0.16 nm, whereas that for the l-2CP complex is 1.94 nm ± 0.01 nm (Figure 7). The radius of gyration for the d-2CP-DehD protein complex was (∼1.90) during the first 50 ps and lower (∼1.94) between 6000–7800 ps of simulation than was for the l-2CP complex (∼1.95). Although the average values for the radii of gyration are insignificant, differing by only 1.55%, their relationship agrees with the relative values found for the RMSD and RMSF values and suggests that the smaller radius of the d-2CP complex also indicates that it is the more stable of the two.

**Figure 8.**
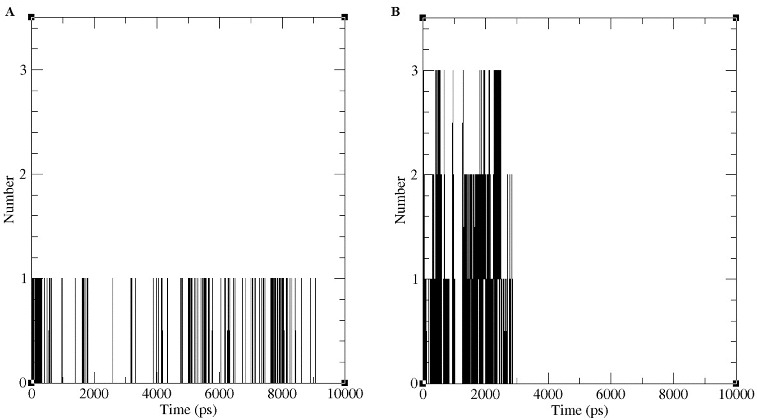
Number of apparent hydrogen bonds vs. time for the d-2CP–DehD (**A**) and l-2CP–DehD (**B**) complexes during the MD simulations.

**Figure 9.**
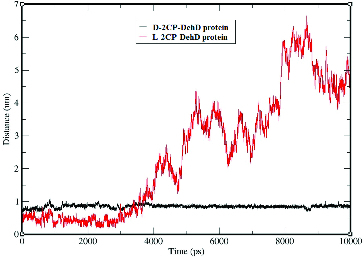
Hydrogen-bond lengths for the d-2CP–DehD and l-2CP–DehD complexes during the MD simulations. d-2CP–DehD (black); l-2CP–DehD (red).

We also identified the number of hydrogen bonds involving d- and l-2CP with DehD. The number of hydrogen bonds (Figure 8) formed by DehD and the two enantiomers were identified after the structures were considered to be stable during the MD run. DehD formed one hydrogen bond with d-2CP of a mean length of 0.85 nm ± 0.05 nm (Figures 8(a) and 9). No hydrogen bond was found for DehD and l-2CP after MD simulation (Figures 8(b) and 9), which may be in part why DehD acts only on d-2CP.[3,32] Charged residues are often associated with local protein flexibility and disorder [33] and may, therefore, decrease protein stability even if they are required to impart structural specificity.[34] Betts and Russell [35] reported that when an arginine is substituted by a lysine, structural stability may be substantially decreased and function may be lost. Much of the long arginine side chain is hydrophobic and consequently is often buried with only the guanidinium group mostly exposed to solvent. This allows the buried area to contain water molecules that may provide additional contacts to arginine quanidinium group.[36] Hence, the hydrogen bond formed by Arg134 and the carboxyl oxygen of d-2CP may be responsible for correctly orienting DehD and d-2CP. This might be responsible for DehD catalysis of d-2CP reported.[3,32] Because the length between the NH of Arg219 and a carboxyl oxygen of l-2CP fluctuated throughout the first 3500 ps of the MD simulation, a stable hydrogen bond appears not to have been formed,[37,38] and the absence of this hydrogen bond may be partially responsible for the inability of DehD to act on l-2CP.[3,32]

During the 9000-ps simulation, only the Arg134-d-2CP hydrogen bond was observed (Figure 8(a)), whereas during the first 2900-ps period, three possible, but non-stable hydrogen bonds were found between DehD and l-2CP (Figure 8(b)). The inability of DehD to form a stable hydrogen bond with l-2CP, which might properly orient the substrate, is consistent with previous experimental data concerning the stereospecificities of various enzymes.[4,32] Tuengler et al. [39] suggested a structural rationale for the enantiomeric substrate preferences of d- and l-lactate dehydrogenase. In l-lactate dehydrogenase, a positional exchange of carboxyl and methyl groups of the substrate by substitutions of a more hydrophobic, uncharged amino-acid residue is responsible for the binding of the carboxyl group and the orientation of the enzyme substrate. In d-lactate dehydrogenase, the carboxyl and methyl groups might be bound to another hydrophobic amino acid residue at a different position, thereby turning the pyruvate molecule around its C=O bond by 180^o^. Additionally, this binding and change of orientation does not affect the catalytic amino acid residue involved in catalysis.[39] Similarly, the carboxyl group of d- and l-2CP is hydrogen bonded to Arg134 and Arg219, respectively in DehD protein. DehD could also be considered to utilize the side chains of their catalytic amino acid residues to bind and turn the orientation of the α-carbon of d-substrates to an l-product.

### A possible reaction mechanism for DehD

DehI catalyse dehalogenation of haloalkanoic acids without the formation of an ester intermediate.[40] Instead, a water molecule directly attacks the α-carbon atom of the substrate to release the halide ion. The structure of the DehI from *Pseudomonas putida* PP3 catalytic centre suggests that Asp189, assisted by Asn114, is responsible for activation of a water molecule as they are adjacent.[9] However, a detailed reaction mechanism for this group of enzymes is generally not well understood, especially the role played by the water molecule,[41] because limited structural information is available.

**Figure 10.**
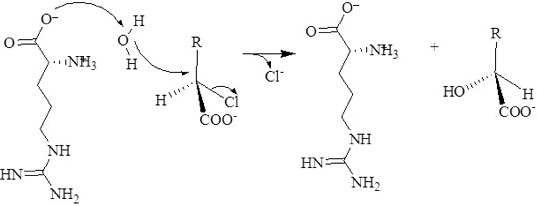
Proposed hydrolytic mechanism for the DehD dehalogenation reaction. Arg16 activates a water molecule for the hydrolytic attack on the carbon atom in position 2, without formation of an ester intermediate.

Docking of a water molecule into the active site of DehD reveals that Arg16 and Val14 of DehD interact with the water and are poised close to each other (Figure 4). Arg16 in DehD has been implicated in the activation of a water molecule necessary for the nucleophilic attack of the carbon in position 2 of d-2-haloalkanoic acid.[10] Polar residues in DehI-type dehalogenases are found in their active sites and may be involved in d-2-haloalkanoic acid dehalogenation [9] (Figure 10). The reaction proceeds with an attack of a water molecule on the carbon in position 2 to displace a hydrogen atom [40] without the formation of an ester intermediate. Arg134 of DehD was found to interact with d-2CP, whilst Val14 and Arg16 residues in DehD interacted with a water molecule. We, therefore, propose that Arg16 acts as the nucleophile and Arg134 is the acid–base catalyst during the DehD hydrolytic reaction. A site-directed mutational study of DehD catalysis may provide more insight into the roles of Val14 and Arg16.

## Conclusions

Our MD simulation indicated that Arg134 forms intermolecular hydrogen bonds with d-2CP and that Arg16 can activate a water molecule to effect a hydrolytic attack on the chiral carbon of d-2CP. Arg134 is proposed to be the key residue specifically involved in the binding of DehD and d-2CP and thus responsible for dictating the stereospecificity of DehD.
